# X-ray crystal structure of the *Escherichia coli* RadD DNA repair protein bound to ADP reveals a novel zinc ribbon domain

**DOI:** 10.1371/journal.pone.0266031

**Published:** 2022-04-28

**Authors:** Miguel A. Osorio Garcia, Kenneth A. Satyshur, Michael M. Cox, James L. Keck

**Affiliations:** 1 Department of Biochemistry, University of Wisconsin, Madison, Madison, WI, United States of America; 2 Department of Biomolecular Chemistry, University of Wisconsin School of Medicine and Public Health, Madison, WI, United States of America; Saint Louis University, UNITED STATES

## Abstract

Genome maintenance is an essential process in all cells. In prokaryotes, the RadD protein is important for survival under conditions that include DNA-damaging radiation. Precisely how RadD participates in genome maintenance remains unclear. Here we present a high-resolution X-ray crystal structure of ADP-bound *Escherichia coli* RadD, revealing a zinc-ribbon element that was not modelled in a previous RadD crystal structure. Insights into the mode of nucleotide binding and additional structure refinement afforded by the new RadD model will help to drive investigations into the activity of RadD as a genome stability and repair factor.

## Introduction

Cells propagate and maintain their genomes by integrating DNA replication, recombination, and repair pathways. Catalysis and regulation of cellular genome maintenance requires the involvement of numerous types of proteins. Many DNA helicase or helicase-like motor proteins are used in DNA metabolism, which requires frequent rearrangement of DNA strands [1, 2].

Several observations point to important roles for the RadD protein, a putative helicase, in processing damaged or branched DNA in *Escherichia coli*. Bioinformatic analysis suggests that *E*. *coli* RadD is a superfamily 2 (SF2) helicase; however, attempts to demonstrate DNA unwinding activity for RadD have failed thus far [3]. The presence of conserved “helicase” motif sequences and the overall structure of RadD place it in the RecQ-like subgroup of the SF2s, which typically contain two RecA-like helicase domains followed by a zinc-binding region and a unique C-terminal domain [4–6]. RadD exhibits *in vitro* ATPase activity that is independent of the presence of DNA [3], which differs from the strongly DNA-dependent ATPase activity observed for RecQ [7]. The *radD* gene, formerly known as *yejH*, has been implicated in double-strand DNA (dsDNA) repair resulting from ionizing radiation and chemical damage in *E*. *coli* [4]. Additional studies have revealed genetic relationships between *radD* and genes encoding SF2 helicases such as *recG* [8] and *recQ* [9], along with *radA* [3], a superfamily 4 (SF4) helicase [10]. RadD binds branched DNA substrates and interacts with single-stranded DNA (ssDNA) binding protein (SSB), consistent with possible RadD involvement in processing branched DNA substrates such as replication forks or DNA repair intermediates [3, 8]. Recent work has revealed that RadD is an accessory protein for RecA, accelerating RecA-mediated DNA strand exchange only in the presence of RecA [11].

A previously-determined structure of RadD revealed structural homology between RadD and RecQ-like helicases, showing that RadD contained a general domain architecture of two RecA-like helicase folds, an intervening zinc-binding region and a variable C-terminal domain [5, 6, 12]. Attempts to model ATP binding through similar homologous structures have been made but direct experimental models have not been reported [5]. Here we present an experimentally determined high-resolution structure of RadD bound to ADP. The structure reveals key differences between apo and ADP-bound RadD, while also revealing a zinc finger that was not modeled in the prior structure. Insights afforded by the presented structure will support future experiments examining the function of RadD within DNA repair in greater detail.

## Results

### RadD-ADP structure

To better understand the structure and function of RadD, we have determined the X-ray crystal structure of nucleotide bound *E*. *coli* RadD. *E*. *coli* RadD was purified, crystallized in the presence of 1 mM ATPγS, and its structure was determined to 2.03-Å resolution using the structure of apo RadD [5] as a molecular replacement search model. The resolution of the diffraction data was an improvement on the prior RadD crystal structure (2.8 Å) [5]. The RadD model was refined with good bond geometry and crystallographic statistics (Table 1). Experimental electron density for the γ-phosphate was notably absent. Thus, ADP was modelled in the RadD active site and we presume that it was produced by slow hydrolysis of ATPγS.

**Table 1:** X-ray crystallographic data collection and structure refinement statistics.

**Data Collection**	
Space group	P1
Unit cell	
a, b, c	44.4, 74.4, 109.4 Å
α, β, γ	84.66°, 80.03°, 82.77°
Resolution	45 to 2.03 Å (2.10 to 2.03 Å)
No. reflections	79347 (5321)
Redundancy	3.3 (2.1)
*I/σI*	10.1 (0.1)
Completeness	98.8% (60.7%)
CC_1/2_	0.983 (0.390)
CC*	0.996 (0.749)
**Refinement**	
Resolution	45–2.03 Å
*R*_*work*_*/R*_free_	0.236/0.269
Root mean square deviation	
Bond length	0.0030 Å
Bond angle	0.6080 Å
Ramachandran statistics	
Residues in core region	96.22
Residues in allowed region	3.78
Residues in disallowed regions	0.00

^*a*^*R* = Σ||*F*_obs_|−|*F*_calc_||/Σ|*F*_obs_|, where the working and free *R* factors are calculated by using the working and free reflection sets, respectively. The free *R* reflections (2.54% of the total) were held aside throughout refinement.

**Fig 1:**
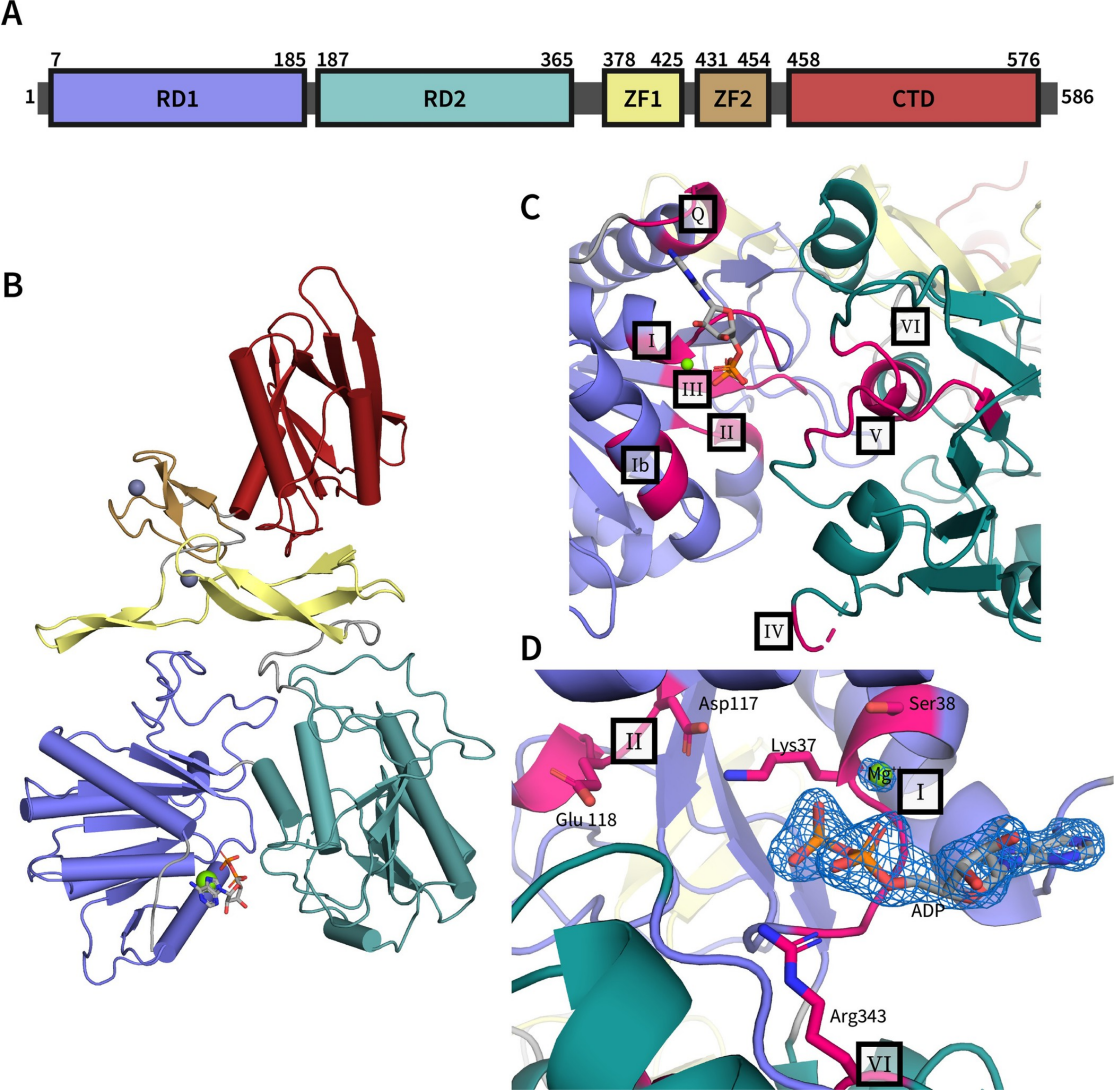
RadD-ADP overall structure and ATPase site. **A)** Schematic map of RadD domains. **B)** Ribbon diagram of RadD-ADP structure RecA-like domain 1 (RD1) in blue, RecA-like domain 2 (RD2) in teal, zinc finger 1 (ZF1) domain in yellow, zinc finger 2 (ZF2) domain in light brown and C-terminal domain (CTD) in red. **C)** RadD ATPase site, with the Q-motif and seven conserved SF2 helicase motifs highlighted in magenta. **D)** View of the ATPase catalytic pocket with 2Fo-Fc electron density of the bound ADP (sticks) and Mg^++^ (green sphere) contoured at 1.6 σ. Motifs I, II, and VI, which are critical for ATP hydrolysis, are highlighted in magenta. Residues important for phosphate coordination and ATP hydrolysis, Lys37 and Ser38 from motif I, Asp117 and Glu118 from motif II, and Arg343 from motif VI, represented as sticks.

With two major exceptions, the ADP-bound RadD was quite similar to the previously published structure of apo RadD [5]. The structural models align with an RMSD of 1.3 Å for all C-alpha atoms, maintaining a similar overall configuration. However, differences were observed in the ATPase active site and in the zinc-binding region. Most notably, the new structure resolved a second zinc-binding motif that was not modelled in the prior apo RadD structure. This observation necessitated a redefinition of the protein domain map for RadD (Fig 1A and 1B).

### ADP occupied ATPase pocket

RadD is a putative helicase that contains seven well conserved SF2 helicase motifs (I, Ia, and II-VI) [4] distributed throughout RecA-like domains 1 and 2 (RD1 and RD2) [5]. These motifs center around the ATP binding pocket, which is present at the interface between RD1 and RD2 (Fig 1C).

In addition to the classical helicase motifs, the ADP-bound RadD structure identified a motif N-terminal to motif I that interacts with the adenine base of ADP (Fig 1C). Similar motifs, called the Q motif or motif 0, have been found in other helicases [12, 13] but the presence of such a motif in RadD has not been noted. These motifs are often structurally similar but with divergent sequences, explaining the previous failure to identify a Q motif in RadD through sequence analysis and the apo RadD structure [4–6]. The RadD Q motif binds ADP through Gln9 and Arg6, where Gln9 hydrogen bonds with N6 and N7 of adenine and Arg6 stabilizes the interaction of ADP by Π-stacking the nitrogenous base. This is quite similar to the adenine-binding motif 0 in RecQ [12].

Similar to other SF2 helicases, RadD motifs I, II, and VI bind the charged phosphates of ATP and likely catalyze pyrophosphate hydrolysis [6, 14]. RadD motif I, also known as the Walker A motif or P-loop [15, 16], primarily binds the α- and β-phosphates through backbone interactions whereas motif I Lys37 is poised to stabilize the presumed γ-phosphate position of ATP (Fig 1D). Ser38 of motif I coordinates a magnesium ion in the structure that in turn stabilizes the diphosphate within ADP. However, motif I is not expected to serve as the magnesium binding site for hydrolysis in RadD. Instead, motif II is predicted to fill this role. Arg343 from motif VI in the RD2 domain of RadD also interacts with the α-phosphate, further stabilizing the ADP interaction (Fig 1D). Motif II of RadD contains Asp117 and Glu118 which in other SF2 helicases catalyze ATP hydrolysis by binding the catalytic magnesium ion that stabilizes the highly-charged ATPase transition state [15, 16]. In the ADP-bound structure, both Asp117 and Glu118 side chains point towards the ADP molecule (Fig 1D).

### Zinc-binding regions

Similar to several other SF2 proteins, RadD contains zinc-binding regions that are proposed to aid with DNA interactions [4]. Sequence analysis from the initial characterization of *radD* reported a putative zinc finger composed of residues Cys434, Cys437, Cys448, and Cys451 [4]. Both *radD* C437A and *radD* K37R mutants are unable to rescue UV sensitivity in *ΔradDΔradA E*. *coli* strains [4], indicating that the putative zinc finger and ATPase are important to the *in vivo* activity of RadD.

**Fig 2:**
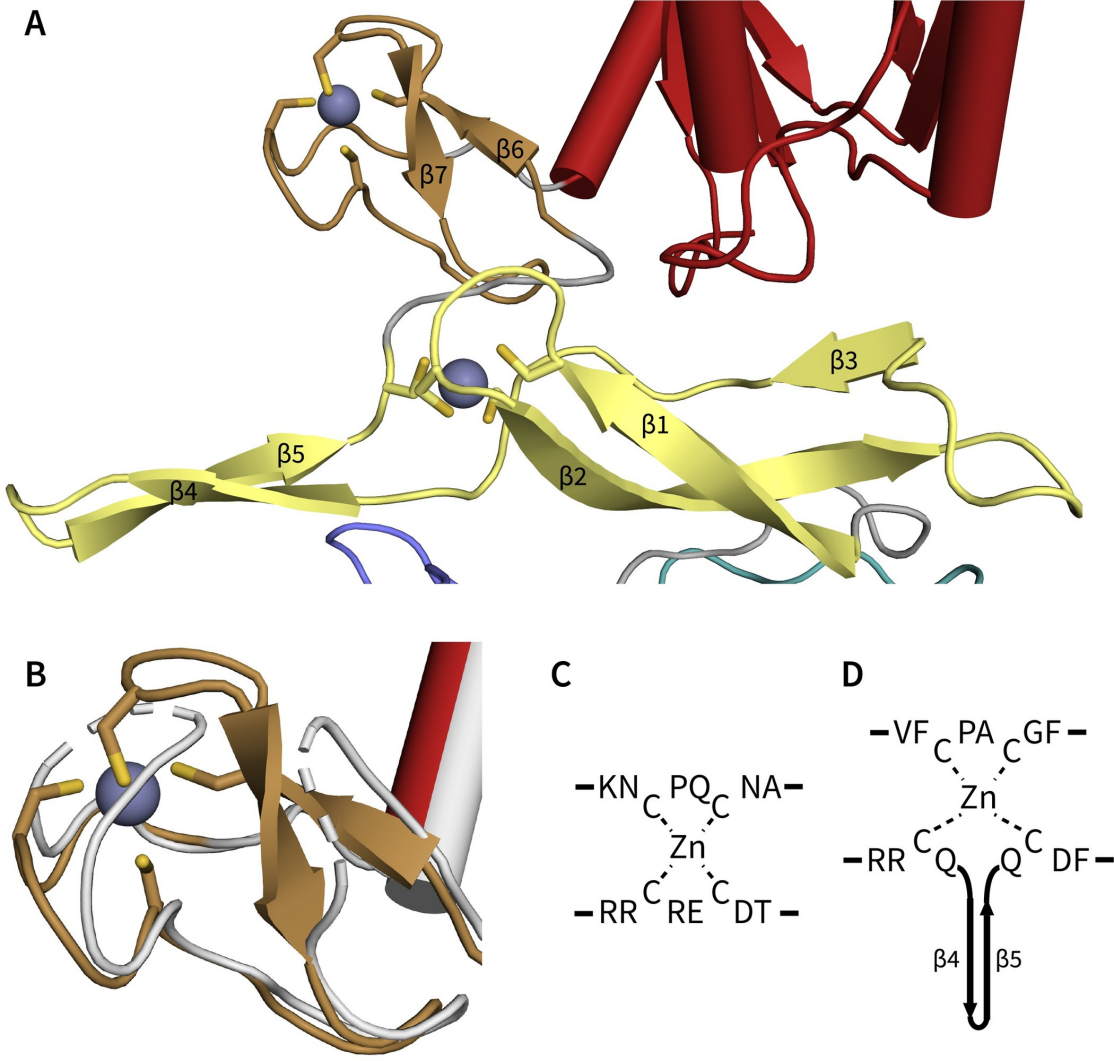
RadD zinc-binding region. **A)** Ribbon diagram of RadD ZF1 in yellow and ZF2 in brown. Zinc ions are depicted as gray spheres coordinated by Cys384, Cys387, Cys411 and Cys425 in ZF1 and Cys434, Cys437, Cys448 and Cys451 in ZF2. Beta strands are numbered. **B)** Alignment comparing apo (grey) and ADP-bound RadD structure. Missing model regions are depicted as dashes. **C)** ZF2 zinc center from residues 432–439 (KNCPQCNA) and 456–463 (RRCRECDT) **D)** ZF1 zinc center from residues 382–389 (VFCPACGF) and 409–427 (RRCQ…QCDIF). Beta strands 4 and 5 of ZF1 are shown as a cartoon representation.

However, the apo structure of RadD identified a zinc finger corresponding to a different location centering around residues Cys384, Cys387, Cys411, and Cys425, relegating the amino acid residue region 433–452 as a flexible linker within the CTD of RadD [5]. The present higher-resolution ADP-bound RadD structure shows unambiguously that RadD contains two zinc fingers, designated as ZF1 and ZF2 (Fig 2A and 2B). ZF1 contains cysteine residues 384, 387, 411 and 425, which comprise a variant zinc ribbon domain [17] composed of two zinc-knuckles formed from the β1-β2 loop and the stem of an antiparallel beta hairpin formed by β4 and β5 (Fig 2A and 2D). ZF2 has a typical zinc-ribbon formation composed of two zinc-knuckles coordinating zinc through cysteine residues 434, 437, 448, and 451 (Fig 2A, 2C and 2D).

## Discussion

We have reported a crystal structure of the *E*. *coli* RadD DNA repair protein, revealing the mechanism of ADP binding and the presence of a novel zinc-binding domain in RadD. The newly identified zinc finger domain (ZF2) was not included in a prior structure although an earlier genetic study predicted its presence in RadD [4, 5]. The role of this second zinc-binding domain is not clear but its importance is supported by genetic studies demonstrating a loss-of-function phenotype when one of the ZF2 zinc-binding cysteine residues is mutated to alanine [4]. Zinc finger motifs have been shown to be important for DNA binding in different RecQ proteins [18–23]. The presence of two zinc fingers in tandem suggests that similar roles may be fulfilled by a multi-zinc-finger DNA binding region in RadD.

Another important factor for DNA binding and helicase activity in many helicases is a β-hairpin or β-wing. These hairpins are composed of a protruding pair of beta strands and are important for DNA binding while functioning as a wedge during strand separation [6, 24–26]. In eukaryoitc RecQ proteins, the β-hairpin is located within a winged-helix domain adjacent to the zinc-binding region and acts as a physical wedge to bind and separate dsDNA junctions [25]. Interestingly, RadD ZF1 includes a prominent β-hairpin made up of β4 and β5 as well as a beta-rich domain making up ZF1 region (Fig 2A and 2C). The presence of a prominent β-hairpin and multiple zinc finger domains clustered in proximity suggests these features may be important for binding of DNA junctions and contribute to DNA repair activity seen *in vivo*. However, it remains to be seen if these features participate in other activities besides DNA binding, such as helicase activity.

**Fig 3:**
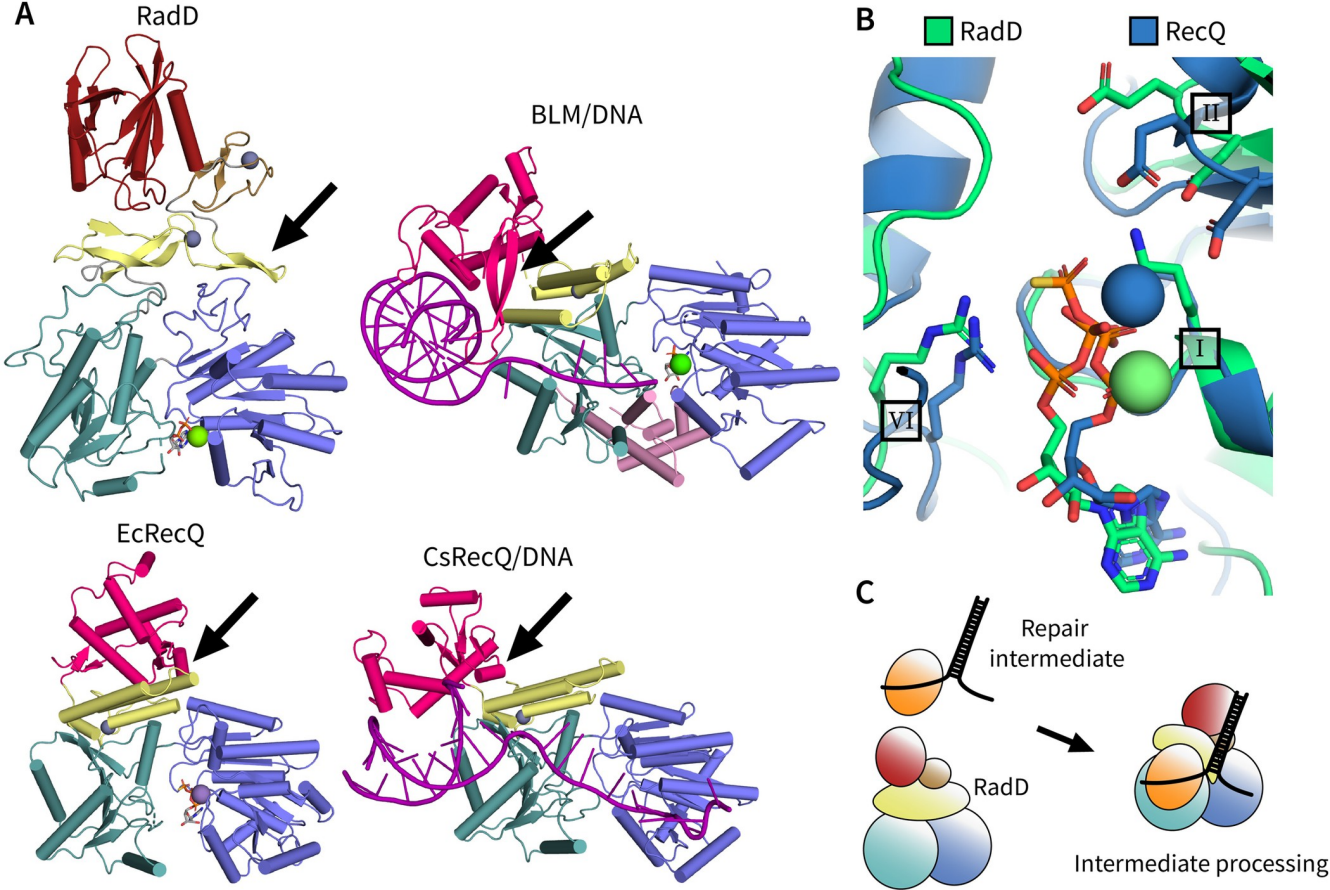
Comparison of RadD and RecQ structures. **A)** Architecture of RadD, human Bloom helicase (BLM) bound to DNA (4O3M), catalytic core of *E*. *coli* RecQ (1OYY), and catalytic core of *C*. *sakazakii* RecQ bound to DNA (4TMU). RecA-like domains in blue and teal, zinc binding regions in yellow, RadD C-terminal domain (CTD) in red, winged helix (WH) domains in bright pink and BLM helicase and ribonuclease D C-terminal (HRDC) domain in light pink. Black arrows point to β-hairpins or DNA interacting loops in RecQs and putative β-hairpin in RadD. **B)** *E coli* RadD and RecQ (1OYY) ATPase pocket aligned to Motif I P-loop. RadD in green and RecQ in blue, residues from helicase motifs I, II, and VI important for ATPase shown as sticks. **C)** Proposed model for RadD activation and conformational changes in the presence of a repair intermediate complex of protein/DNA substrates.

Although the roles of RadD remain undefined, the similarities between RadD and RecQ-like proteins point to RadD functioning as an accessory protein in DNA/protein repair intermediates. RadD shares a similar domain architecture with *E*. *coli* RecQ helicase core as shown in Fig 3, differing primarily in the presence of a C-terminal winged helix domain in RecQ and the unique CTD in RadD. *E*. *coli* RecQ lacks a β-hairpin DNA unwinding wedge whereas human RecQ helicases such as the Bloom syndrome protein (BLM) employ a β-hairpin located in their WH domain, which is closer to the RadD architecture (Fig 3A). Bacterial RecQ undergoes a large rearrangement of its C-terminal domains when binding DNA where the winged helix domain closes on the remainder of the protein to clamp the ss/ds DNA junction [22]. RadD might function in a similar manner where the CTD or beta strand rich zinc binding regions are rearranged to interact with forked DNA and/or protein partners (Fig 3C).

The ADP-bound RadD structure shows a similar nucleotide binding mechanism when compared to other SF2 helicases. The main helicase folds responsible for ATP binding and hydrolysis are poised in a manner typical for active SF2 helicases, such as the positioning of motifs Q, I, II, and VI (Figs 1 and 3B). The catalytic lysine and arginine from motifs I and VI, respectively, appear poised to stabilize γ-phosphate hydrolysis in the apo form of the enzyme, similar to RecQ (Fig 3B). The remaining helicase motifs are important in other RecQ-like proteins and SF2s as elements that coordinate DNA binding with ATPase activity [6]. The glutamate and aspartate in motif II of RadD are further from the ATPase site when compared to RecQ (Fig 3B). These residues coordinate a catalytic magnesium ion in helicases (Fig 3B). RecQ links DNA binding to ATPase activity through motif II as it is directly connected to an aromatic rich region of RecQ that interacts with ssDNA, causing conformational rearrangements of the ATPase site that facilitate hydrolysis [22]. RadD is missing this aromatic rich region which may explain its decoupling of direct DNA binding and ATPase. However, a recent study shows RadD is a RecA accessory protein that accelerates RecA-mediated DNA strand exchange [11]. As such, RadD may well carry out this function utilizing a helicase-like mechanism. The requirement for a RecA interaction may eventually help explain some of the structural distinctions between RadD and other members of this helicase family.

The insights into the structure of RadD/ADP and the possible links to structural and functional assessments of RadD activity afforded by the new structure presented here could help to dissect the roles of RadD in DNA repair. Further studies linking the full structure of RadD to its biochemical and *in vivo* activities would greatly aid in the understanding of the role of RadD in DNA metabolism.

## Methods

### Protein purification

RadD was purified to >95% purity by gel analysis as previously reported [3]. Briefly, *E*. *coli* strain STL2669 (DE3) was transformed with pEAW724 [3] containing the open reading frame of *E*. *coli radD* and induced over 3 hours with 1 mM IPTG. Cells were pelleted and resuspended in lysis buffer (25% (w/v) sucrose, 250 mm Tris chloride (pH 7.7), 7 mm EDTA, 1 μm pepstatin, 1 μm leupeptin, 1 μm E-64) and lysed using 5mg/ml lysozyme and sonication. Lysate was clarified by centrifugation and RadD was precipitated from supernatant by the addition of solid (NH_4_)_2_SO_4_. The (NH_4_)_2_SO_4_ pellet was resuspended in R-Buffer (20 mm Tris chloride (pH 7.7), 1 mm EDTA, 10% glycerol) + 1 M (NH_4_)_2_SO_4_ and loaded on to a butyl-Sepharose column. RadD was eluted with a gradient of 1 to 0 molar (NH_4_)_2_SO_4_ and dialyzed into 20 mm phosphate (pH 7.0), 200 mM KCl, 1 mm EDTA, and 10% glycerol. Dialyzed RadD was then applied to a ceramic hydroxyapatite column and collected in the wash. Fractions containing RadD were pooled and dialyzed into R-Buffer + 200 mM KCl and ran on both source-15S and source-15Q. RadD flowed through both columns while contaminants remained bound. The purified protein was >95% pure by gel, flash frozen and stored at -80°C. Protein concentration was determined using an ε_280_ of 5.59 × 10^4^ M^−1^cm^−1^ [3].

### RadD crystallization

RadD (6 mg/ml) was dialyzed into minimal buffer containing 20 mM Tris-HCl pH 7.7, 200 mM KCl, 1 mM DTT, 1 mM MgCl_2_ and mixed with 1 mM ATPγS. RadD was crystalized by mixing 1 μl of protein with 1 μl of mother liquor (150 mM NaSCN, 18% PEG 3350) in a hanging drop vapor diffusion experiment incubated at 4°C. Crystals were harvested after ~2 weeks by adding ethylene glycol to 25% (cryoprotectant) and freezing in liquid nitrogen.

### Data processing and structure determination

Data were indexed and scaled using HKL2000 [27] and phased by molecular replacement using the apo RadD structure (PDB: 6JDE) as a search model with PHASER [28]. Iterative rounds of manual building and refinement were conducted using COOT [29] and PHENIX [30]. Structure deposited under PDB ID 7R7J.
